# Impact of low preoperative appendicular skeletal muscle mass on postoperative complications and short-term outcomes in liver transplant recipients: a propensity score-matched retrospective study

**DOI:** 10.3389/fsurg.2025.1671709

**Published:** 2025-09-26

**Authors:** Jing Xu, Qian Wu, Xiaofeng Xu, Feihong Weng, Tao Lv, Jian Yang, Shouping Wang, Rui Li, Chengbo Ai, Gang Xu, Lvnan Yan, Jiayin Yang

**Affiliations:** 1 Center for Organ Transplantation and Liver Transplantation, West China Hospital, Sichuan University, Chengdu, China; 2 West China School of Nursing, Sichuan University, Chengdu, China; 3 West China School of Clinical Medicine, Sichuan University, Chengdu, China; 4 Department of Pediatric Surgery, Children’s Hospital of Fudan University, Shanghai, China; 5 Department of Pediatric Surgery, West China Hospital/West China School of Medicine, Sichuan University, Chengdu, China

**Keywords:** low appendicular skeletal muscle mass, liver transplant, postoperative complications, short-term prognosis, propensity score matching

## Abstract

**Background:**

Low preoperative appendicular skeletal muscle mass (ASM) is common in liver transplantation (LT) recipients and may be linked to adverse postoperative outcomes. This study explored the relationship between preoperative ASM and short-term postoperative outcomes, including perioperative inflammation.

**Methods:**

We retrospectively analyzed 653 LT patients at West China Hospital from 2015 to 2022. ASM index (ASM/H^2^) was calculated using Asian Working Group for Sarcopenia (AWGS) standards. Patients were classified into low and non-low ASM groups by sex-specific cutoffs. Propensity score matching (PSM, 1:1) was used to control for confounding. Associations with complications, inflammatory markers, and survival were evaluated using multivariate logistic and Cox regression. The predictive performance was evaluated using receiver operating characteristic (ROC) curves.

**Results:**

After PSM, 84 matched pairs were analyzed. On postoperative days 1 and 3, the low ASM group had significantly higher neutrophils, NLR, MLR, and NMR (*P* < 0.05), and lower lymphocyte and platelet counts. This group also showed increased early complications, including pulmonary infection, pleural effusion, and intra-abdominal bleeding (in-hospital mortality: 9.52% vs. 1.19%, *P* = 0.040). Low ASM independently predicted complications (OR = 6.61, 95% CI: 3.08–14.21) and worse overall survival (HR = 2.25, 95% CI: 1.41–3.57). Predictive models including ASM achieved high accuracy (AUC = 0.80 for complications; AUC = 0.75 for survival).

**Conclusions:**

Low preoperative ASM is an independent risk factor for inflammation, complications, and poorer survival after LT. ASM screening may improve early risk stratification and guide perioperative care.

## 1 Introduction

Muscle wasting, marked by a gradual loss of skeletal muscle and a corresponding decline in function, is a common pathological condition affecting both the elderly and those with chronic illnesses (1). Several international bodies have proposed the definitions and diagnostic criteria for this condition (2–4). Given the anatomical and metabolic variations across different ethnic groups, the assessment of muscle mass in Asian populations typically adheres to the guidelines established by the Asian Working Group for Sarcopenia (AWGS) (5). The AWGS advocates the use of appendicular skeletal muscle mass adjusted for height (ASM/Height^2^), a metric that has demonstrated a superior ability to predict functional deterioration and adverse clinical outcomes associated with low muscle mass (6).

Although sarcopenia has been extensively investigated in the general population, research concerning its impact on patients with chronic liver disease and those awaiting liver transplantation (LT) remains limited. Existing evidence suggests that a combination of factors, including reduced nutrient intake, increased metabolism, altered amino acid profiles, endotoxemia, prolonged immobility, and physical deconditioning, contribute to diminished skeletal muscle synthesis and increased breakdown, thereby accelerating muscle loss (7). In individuals awaiting LT, the presence of widespread nutritional, metabolic, and biochemical disturbances further exacerbates the imbalance between protein synthesis and degradation, ultimately leading to secondary sarcopenia (8, 9). A recent meta-analysis highlighted that the prevalence of muscle wasting among patients with chronic liver disease ranges from 40% to 70%, with significant variations observed across different ethnicities (10).

Previous studies have established a strong association between reduced muscle mass and increased mortality during the waiting period, intraoperative phase, and postoperative course of LT (11–13). Consequently, systematic preoperative evaluation of skeletal muscle mass has been increasingly incorporated into perioperative management recommendations. The North American expert consensus on sarcopenia in LT strongly advocates routine muscle status assessment in patients with cirrhosis prior to transplantation and recommends individualized preoperative interventions involving exercise and nutritional support to reduce postoperative infection rates, shorten hospital stays, and improve overall outcomes (14). Various techniques are currently available to assess muscle mass prior to LT, including magnetic resonance imaging (MRI), dual-energy x-ray absorptiometry (DXA), ultrasonography, bioelectrical impedance analysis (BIA), and the D3-creatine dilution method (15–22). However, many of these methods are complex or limited by the unique pathophysiological conditions of liver disease. By contrast, the AWGS-recommended prediction equation for ASM, which incorporates body weight, height, sex, and age, is a practical and scalable tool suitable for large-scale epidemiological studies.

Although preliminary studies have explored the association between sarcopenia and the postoperative outcomes in LT, large-scale cohort studies employing clinically applicable predictive equations are still scarce. Moreover, the potential relationship between preoperative muscle wasting and the postoperative inflammatory response in transplant recipients is yet to be fully clarified. Our center boasts an established LT program with one of the highest patient volumes in China, ensuring data consistency and minimal heterogeneity. Leveraging these clinical gaps and our institutional strengths, this study aimed to: (1) ascertain the impact of preoperative muscle mass on postoperative outcomes in liver transplant recipients and (2) investigate whether changes in inflammatory status occur in sarcopenic patients undergoing LT.

## 2 Methods

### 2.1 Data source and study population

Data were obtained from the Clinical Big Data Search Engine Database of the West China Hospital, Sichuan University (http://hxdmc.cn). A total of 653 patients who underwent LT between January 2015 and December 2022 were retrospectively analyzed. The exclusion criteria were as follows: (1) patients under 18 years of age, (2) substantial missing data that prevented the calculation of muscle mass or determination of outcomes, (3) the presence of other malignant solid tumors (e.g., extrahepatic metastasis), and (4) patients who received combined organ transplantation (e.g., liver-kidney transplantation).

This was a retrospective study. All procedures were performed in accordance with the ethical standards set forth by the Ethics Committee of West China Hospital and national regulations as well as the 1964 Declaration of Helsinki and its subsequent amendments. The requirement for informed consent was waived by the ethics committee because the study did not involve direct patient intervention.

### 2.2 Measurement of muscle mass

The appendicular skeletal muscle mass (ASM) was estimated using the following formula proposed by the Asian Working Group for Sarcopenia (2019): ASM/Ht^2^ = 0.193 × weight (kg) + 0.107 × height (cm) − 4.157 × sex (male = 1, female = 2) − 0.037 × age (years) − 2.631. The ASM index was subsequently calculated as ASM divided by the height squared (ASM/Ht^2^). Using threshold values of 6.88 kg/m^2^ for men and 5.69 kg/m^2^ for women, patients were categorized into either the low muscle mass or normal muscle mass groups.

### 2.3 Study outcomes

The clinical outcomes were divided into short-term and long-term categories. The short-term outcomes included early postoperative complications (primary endpoint), length of hospital stay, need for respiratory support, unplanned ICU admission, in-hospital mortality, and reoperation. The length of stay was defined as the number of days between the date of surgery and discharge. Unplanned ICU admission was defined as clinical deterioration after surgery necessitating transfer from the ward to the ICU. Respiratory support was indicated by either failure to extubate postoperatively or reintubation because of clinical worsening. In-hospital mortality was defined as death prior to hospital discharge.

Overall survival (OS) was defined as the time elapsed from LT until death from disease or the date of the last follow-up. All patients underwent standardized postoperative follow-up, which included review of outpatient records, inpatient revisit records, and telephone interviews. The follow-up period was December 31, 2024, with a minimum follow-up window of 12 months. Survival outcome assessments adhered to the guidelines published by the Chinese Society of Clinical Oncology (CSCO) to maintain consistency and data completeness.

### 2.4 Clinical and pathological parameters

The clinical variables assessed included preoperative characteristics [sex, age, body mass index (BMI), history of hypertension, history of diabetes, prior retransplantation, primary diagnosis, surgical technique, MELD score, Child–Pugh score, biliary complications, and alpha-fetoprotein (AFP) level]. Intraoperative variables (graft weight, cold ischemia time, intraoperative blood loss, volume of intraoperative transfusion, and duration of surgery).

Peripheral blood samples were collected preoperatively and on days 1, 3, and 7 postoperatively. Laboratory data included neutrophil, monocyte, lymphocyte, white blood cell, and platelet counts. The following inflammatory indices were subsequently calculated: neutrophil-to-lymphocyte ratio (NLR), platelet-to-lymphocyte ratio (PLR), monocyte-to-lymphocyte ratio (MLR), neutrophil-to-monocyte ratio (NMR), and systemic immune-inflammation index (SII), calculated as the product of platelet and neutrophil counts divided by lymphocyte count.

### 2.5 Statistical analysis

Statistical analyses were performed using the R software (version 4.2.0). The Shapiro–Wilk test was used to ascertain the normality of continuous variables. Variables exhibiting a normal distribution were reported as mean ± standard deviation (SD) and compared using independent-samples *t*-tests. Conversely, non-normally distributed variables are presented as medians (interquartile range, IQR) and analyzed using the Mann–Whitney *U* test. Categorical variables are summarized as frequencies and percentages, and group comparisons were made using the chi-square test or Fisher's exact test, depending on the suitability of each.

To mitigate potential confounding, 1:1 nearest-neighbor propensity score matching (PSM) was implemented using a caliper width of 0.02. The covariates included in the matching process were age, sex, BMI, comorbidities, etiology of liver disease, surgical type, MELD score, and Child–Pugh classification. An adequate covariate balance was deemed to be achieved when the standardized mean difference (SMD) between the groups was less than 0.1. Univariate and multivariate logistic regression analyses were performed to examine the relationship between low preoperative muscle mass and early postoperative complications. Kaplan–Meier curves were used to estimate cumulative overall survival (OS), and differences between groups were assessed using the log-rank test. Cox proportional hazards regression analysis was conducted to identify predictors of OS with hazard ratios (HRs) and their corresponding 95% confidence intervals (CIs). The predictive accuracy of the models was evaluated using receiver operating characteristic (ROC) curves and the associated area under the curve (AUC). All statistical tests were two-sided, and statistical significance was set at *P* < 0.05.

## 3 Results

### 3.1 Baseline characteristics of patients

**Figure 1:**
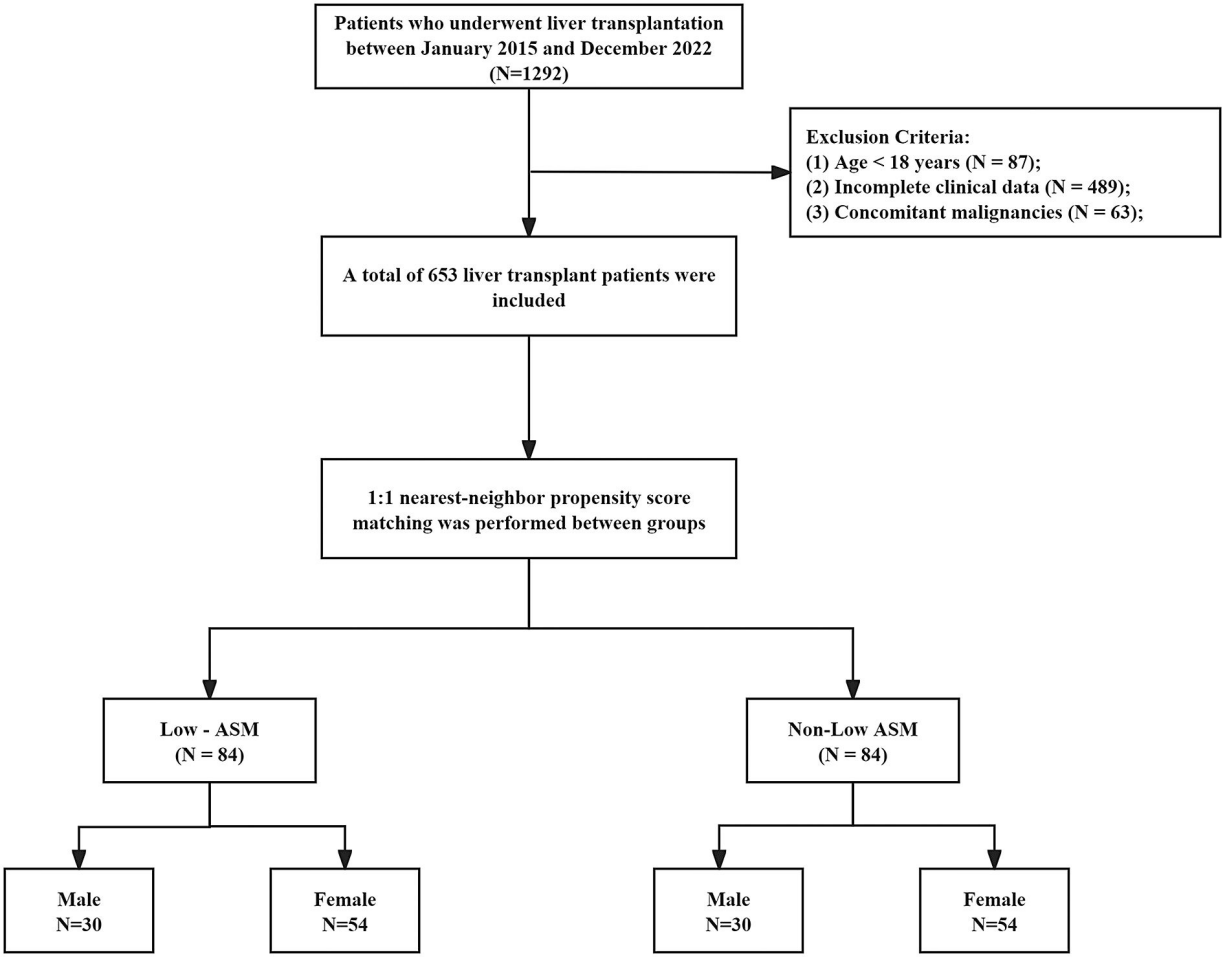
Flowchart of inclusion and exclusion of study population.

A total of 653 patients who underwent LT at West China Hospital, Sichuan University, between January 2015 and December 2022 were included in this study. The patient selection process is illustrated in Figure 1. The mean age of the cohort was 50 ± 10.23 years, with 78.7% male and 21.3% female patients. Based on the ASM evaluation, 84 patients (12.7%) were classified as having low preoperative muscle mass. Compared with patients in the normal muscle mass group, those in the low muscle mass group had a higher prevalence of liver malignancy (38.1% vs. 32.1%, *P* < 0.001), and exhibited significantly elevated levels of peripheral blood inflammatory markers prior to transplantation, including neutrophil count (10.78 ± 6.24 vs. 7.13 ± 4.25, *P* < 0.001), white blood cell count (1,059.34 ± 1,542.43 vs. 862.81 ± 1,037.72, *P* < 0.001), NLR (39.72 ± 27.83 vs. 15.98 ± 13.47, *P* < 0.001), MLR (1.52 ± 1.45 vs. 1.01 ± 0.83, *P* = 0.002), NMR (31.88 ± 19.26 vs. 18.04 ± 13.21, *P* < 0.001), and SII (2,355.02 ± 4,309.20 vs. 912.42 ± 1,045.32, *P* < 0.001). Conversely, lymphocyte count (0.33 ± 0.21 vs. 0.55 ± 0.36, *P* < 0.001) and PLR (167.60 ± 162.28 vs. 211.96 ± 132.92, *P* = 0.006) were significantly lower in the low muscle mass group. To account for potential confounders, propensity score matching (PSM) was applied using a 1:1 nearest-neighbor approach, yielding 84 matched pairs. Post-matching analysis confirmed balanced baseline characteristics—age, sex, BMI, comorbidities, liver disease etiology, and surgical procedure—between groups, with no statistically significant disparities (see Table 1).

**Table 1:** Baseline characteristics before and after PSM.

Characteristic	Original cohort			Matched cohort		
	Low—ASM (*N* = 84)	Non-low ASM (*N* = 569)	*P*-value	Low—ASM (*N* = 84)	Non-low ASM (*N* = 84)	*P*-value
Age, mean (SD), years	50.9 ± 12.6	49.9 ± 9.8	0.485	50.9 ± 12.6	48.9 ± 10.9	0.284
Sex, *n* (%)			<0.001			1.000
Male	30 (35.7%)	484 (85.1%)		30 (35.7%)	30 (35.7%)	
Female	54 (64.3%)	85 (14.9%)		54 (64.3%)	54 (64.3%)	
BMI, mean (SD), kg/m^2^	18.4 ± 2.1	23.9 ± 2.9	<0.001	18.4 ± 2.1	24.1 ± 2.6	<0.001
Total Bilirubin, mean (SD)	175.3 ± 174.3	142.9 ± 170.1	0.105	175.3 ± 174.3	169.3 ± 188.1	0.830
Hypertention			0.012			0.129
No	84 (100%)	529 (93.0%)		84 (100%)	80 (95.2%)	
Yes	0 (0%)	40 (7.0%)		0 (0%)	4 (4.8%)	
Diabetes			0.061			<0.001
No	81 (96.4%)	513 (90.2%)		81 (96.4%)	57 (67.9%)	
Yes	3 (3.6%)	56 (9.8%)		3 (3.6%)	27 (32.1%)	
Second liver transplant			0.026			0.477
No	82 (97.6%)	567 (99.6%)		82 (97.6%)	84 (100.0%)	
Yes	2 (2.4%)	2 (0.4%)		2 (2.4%)	0 (0.0%)	
Diagnostic			<0.001			0.026
Malignant liver tumor	32 (38.1%)	310 (54.5%)		32 (38.1%)	52 (61.9%)	
Post-hepatitis cirrhosis with decompensation	27 (32.1%)	207 (36.4%)		27 (32.1%)	16 (19.0%)	
Metabolic liver disease	2 (2.4%)	3 (0.5%)		2 (2.4%)	1 (1.2%)	
Alcoholic liver disease	2 (2.4%)	22 (3.9%)		2 (2.4%)	1 (1.2%)	
Others	21 (25.0%)	27 (4.7%)		21 (25.0%)	14 (16.7%)	
Surgical procedure			0.007			0.721
Living related LT	7 (8.3%)	54 (9.5%)		7 (8.3%)	9 (10.7%)	
Allogeneic LT	73 (86.9%)	511 (89.8%)		73 (86.9%)	73 (86.9%)	
Homologous split LT	4 (4.8%)	4 (0.7%)		4 (4.8%)	2 (2.4%)	
Meld Score			0.258			0.841
≤10	21 (25.0%)	169 (29.7%)		21 (25.0%)	21 (25.0%)	
11–18	23 (27.4%)	169 (29.7%)		23 (27.4%)	23 (27.4%)	
19–24	18 (21.4%)	76 (13.4%)		18 (21.4%)	14 (16.7%)	
≥25	22 (26.2%)	155 (27.2%)		22 (26.2%)	26 (31.0%)	
Child-pugh Score			0.989			0.822
A	2 (2.4%)	14 (2.5%)		2 (2.4%)	2 (2.4%)	
B	40 (47.6%)	266 (46.7%)		40 (47.6%)	35 (41.7%)	
C	42 (50.0%)	289 (50.8%)		42 (50.0%)	47 (56.0%)	
Pre-AFP, mean (SD)^a^	535.3 ± 3,372.6	265.7 ± 3,208.5	0.475	535.3 ± 3,372.6	144.0 ± 355.0	0.292
Pre-Neutrophil, mean (SD)^a^	10.78 ± 6.24	7.13 ± 4.25	<0.001	7.00 ± 4.41	7.67 ± 4.40	0.326
Pre-Monocyte, mean (SD)^a^	0.44 ± 0.37	0.48 ± 0.33	0.338	0.47 ± 0.36	0.53 ± 0.40	0.305
Pre-Lymphocyte, mean (SD)^a^	0.33 ± 0.21	0.55 ± 0.36	<0.001	0.54 ± 0.32	0.62 ± 0.28	0.072
Pre-Leucocyte, mean (SD)^a^	1,059.34 ± 1,542.43	862.81 ± 1,037.72	<0.001	8.13 ± 4.84	8.90 ± 4.84	0.304
Pre-Platelet, mean (SD)^a^	49.80 ± 46.54	58.49 ± 41.41	0.078	63.56 ± 55.83	58.82 ± 36.03	0.514
Pre-NLR, mean (SD)^a^	39.72 ± 27.83	15.98 ± 13.47	<0.001	15.96 ± 12.78	13.75 ± 9.08	0.198
Pre-PLR, mean (SD)^a^	167.60 ± 162.28	211.96 ± 132.92	0.006	132.39 ± 124.63	108.79 ± 74.55	0.138
Pre-MLR, mean (SD)^a^	1.52 ± 1.45	1.01 ± 0.83	0.002	0.97 ± 0.86	0.92 ± 0.66	0.665
Pre-NMR, mean (SD)^a^	31.88 ± 19.26	18.04 ± 13.21	<0.001	20.17 ± 18.85	16.74 ± 9.41	0.138
Pre-SII, mean (SD)^a^	2,355.02 ± 4,309.20	912.42 ± 1,045.32	<0.001	1,059.34 ± 1,542.43	862.81 ± 1,037.72	0.334

Data are presented as mean ± SD or median (IQR) as appropriate. Comparisons were made using the independent *t*-test or Mann–Whitney *U* test for continuous variables, and the chi-square or Fisher's exact test for categorical variables.

a Peripheral blood samples were collected within 1 week before surgery.

### 3.2 Correlation between preoperative low muscle mass and postoperative inflammatory markers

After PSM, there were no statistically significant differences in preoperative peripheral blood inflammatory markers, including neutrophil count, monocyte count, lymphocyte count, NLR, MLR, NMR, PLR, and SIIbetween, between the two groups.

**Figure 2:**
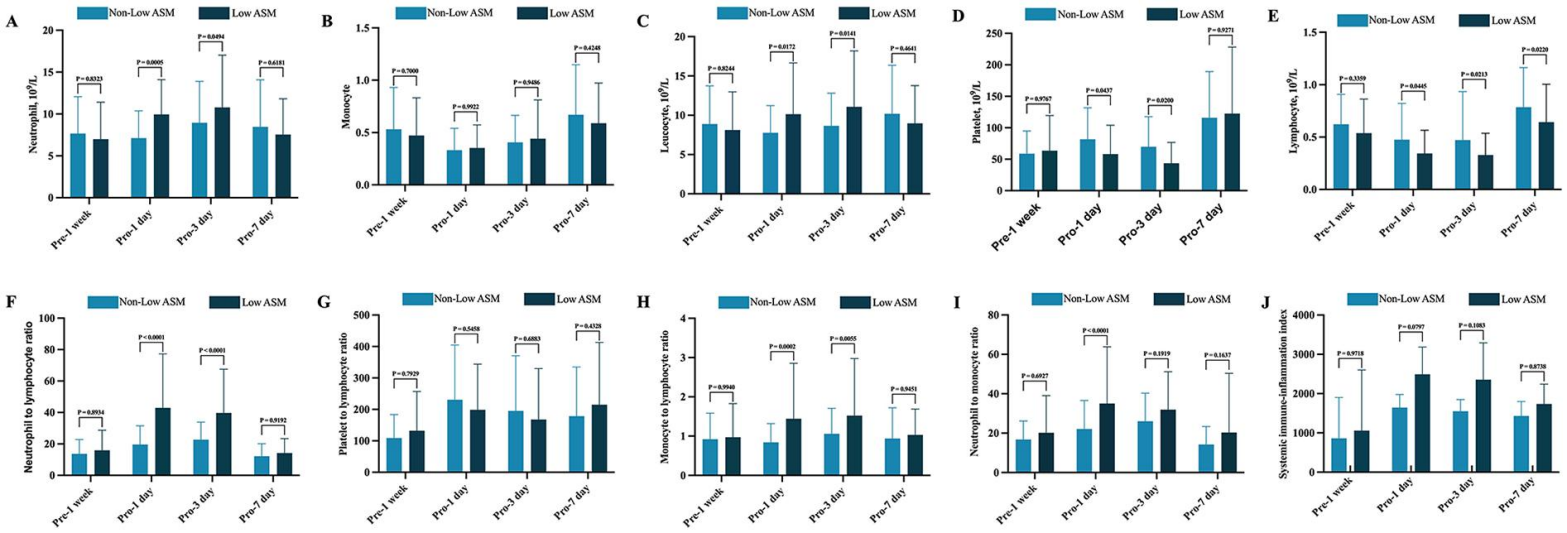
Dynamic perioperative changes in peripheral blood inflammatory markers between patients with and without Low preoperative ASM. **(A)** Neutrophil; **(B)** Monocyte; **(C)** Lymphocyte; **(D)** Leukocyte; **(E)** Platelet; **(F)** NLR (neutrophil-to-lymphocyte ratio); **(G)** PLR (platelet-to-lymphocyte ratio); **(H)** MLR (monocyte-to-lymphocyte ratio); **(I)** NMR (neutrophil-to-monocyte ratio); **(J)** SII (product of platelet count and neutrophil count divided by lymphocyte count). All results were analyzed using the following statistical methods. Normally distributed data were expressed as mean ± standard deviation (SD) and compared using the independent samples *t*-test. Non-normally distributed data are expressed as median and interquartile range (IQR) and analyzed using the Mann–Whitney *U* test.

However, dynamic postoperative hematological monitoring indicated that patients classified in the low ASM group exhibited markedly elevated levels of neutrophils, NLR, MLR, and NMR on postoperative days 1 and 3 compared with those in the non-ASM group. This finding suggests a robust inflammatory response in patients with reduced muscle mass. Conversely, lymphocyte and platelet counts were considerably lower in the low muscle mass group, potentially indicating a diminished immune capacity (see Figure 2).

### 3.3 Correlation between low preoperative ASM and short-term clinical outcomes

Of the 653 liver transplant recipients involved in the study, 180 experienced early postoperative complications. After PSM, the frequency of these early complications was notably higher in the group with low preoperative appendicular skeletal muscle mass than that in the control group. These complications encompassed the requirement for respiratory support, unplanned intensive care unit (ICU) admission, and in-hospital mortality (Table 2). Specifically, in-hospital mortality was significantly higher in the low muscle mass group (9.52% vs. 1.19%, *P* = 0.040). Examining specific complications, the low muscle mass group presented with a significantly higher incidence of pulmonary infection (16.67% vs. 2.38%, *P* = 0.002), pleural effusion (13.10% vs. 3.57%, *P* = 0.026), and intra-abdominal bleeding (11.90% vs. 1.19%, *P* = 0.005). Furthermore, other complications, such as biliary stricture, urinary tract infection, and gastrointestinal bleeding, were observed more frequently in the low muscle mass group, while some of these did not achieve statistical significance; they demonstrated a discernible upward trend.

**Table 2:** Comparison of postoperative complications and outcomes between patients with and without low preoperative ASM before and after PSM.

Factors	Original cohort			Matched cohort		
	Low—ASM (*N* = 84)	Non-low ASM (*N* = 569)	*P*-value	Low—ASM (*N* = 84)	Non-low ASM (*N* = 84)	*P*-value
Early complications						
No	31 (36.90)	442 (77.68)	<.001	31 (36.90)	68 (80.95)	<.001
Yes	53 (63.10)	127 (22.32)		53 (63.10)	16 (19.05)	
Respiratory support			0.401			0.342
No	64 (76.19)	456 (80.14)		64 (76.19)	69 (82.14)	
Yes	20 (23.81)	113 (19.86)		20 (23.81)	15 (17.86)	
Unplanned transfer to ICU			0.551			0.726
No	61 (72.62)	395 (69.42)		61 (72.62)	63 (75.00)	
Yes	23 (27.38)	174 (30.58)		23 (27.38)	21 (25.00)	
In-hospital death			0.002			0.040
No	76 (90.48)	555 (97.54)		76 (90.48)	83 (98.81)	
Yes	8 (9.52)	14 (2.46)		8 (9.52)	1 (1.19)	
Length of stay	18.07 ± 13.30	19.47 ± 16.03	0.445	22.36 ± 16.47	18.74 ± 14.69	0.135
Types of complications						
Wound infections			0.009			0.364
No	80 (95.24)	565 (99.30)		80 (95.24)	83 (98.81)	
Yes	4 (4.76)	4 (0.70)		4 (4.76)	1 (1.19)	
Pulmonary infection			<.001			0.002
No	70 (83.33)	539 (94.73)		70 (83.33)	82 (97.62)	
Yes	14 (16.67)	30 (5.27)		14 (16.67)	2 (2.38)	
Pleural effusion			0.053			0.026
No	73 (86.90)	529 (92.97)		73 (86.90)	81 (96.43)	
Yes	11 (13.10)	40 (7.03)		11 (13.10)	3 (3.57)	
Intra-abdominal bleeding			<.001			0.005
No	74 (88.10)	567 (99.65)		74 (88.10)	83 (98.81)	
Yes	10 (11.90)	2 (0.35)		10 (11.90)	1 (1.19)	
Biliary stasis			<.001			0.122
No	78 (92.86)	566 (99.47)		78 (92.86)	83 (98.81)	
Yes	6 (7.14)	3 (0.53)		6 (7.14)	1 (1.19)	
Liver failure			0.083			0.477
No	82 (97.62)	567 (99.65)		82 (97.62)	84 (100.00)	
Yes	2 (2.38)	2 (0.35)		2 (2.38)	0 (0.00)	
Bile leakage			1.000			1.000
No	82 (97.62)	557 (97.89)		82 (97.62)	81 (96.43)	
Yes	2 (2.38)	12 (2.11)		2 (2.38)	3 (3.57)	
Biliary stricture			0.012			0.349
No	77 (91.67)	555 (97.54)		77 (91.67)	80 (95.24)	
Yes	7 (8.33)	14 (2.46)		7 (8.33)	4 (4.76)	
Urinary tract infection			0.005			0.440
No	79 (94.05)	563 (98.95)		79 (94.05)	82 (97.62)	
Yes	5 (5.95)	6 (1.05)		5 (5.95)	2 (2.38)	
Gastrointestinal bleeding			0.059			0.070
No	77 (91.67)	550 (96.66)		77 (91.67)	83 (98.81)	
Yes	7 (8.33)	19 (3.34)		7 (8.33)	1 (1.19)	
Intestinal obstruction			0.339			1.000
No	83 (98.81)	567 (99.65)		83 (98.81)	83 (98.81)	
Yes	1 (1.19)	2 (0.35)		1 (1.19)	1 (1.19)	

Multivariate logistic regression analysis confirmed that low preoperative appendicular skeletal muscle mass was an independent risk factor for early postoperative complications (OR =  6.61, 95% CI: 3.08–14.21, *P* < 0.001). This association persisted even after accounting for potential confounding variables including sex, age, and baseline comorbidities. In addition to ASM, decompensated cirrhosis (OR = 1.55, *P* = 0.031) and intraoperative blood loss >1,000 ml (OR = 1.54, *P* = 0.027) were also identified as significant predictors of postoperative complications (Table 3).

**Table 3:** Univariate and multivariate logistic regression for early postoperative complications after PSM.

Factors	Univariable analysis		Multivariate analysis	
	OR (95% CI)	*P*-value	OR (95% CI)	*P*-value
Age				
<60	1.00 (Reference)			
≥60	1.35 (0.88–2.08)	0.166		
Sex				
Male	1.00 (Reference)		1.00 (Reference)	
Female	2.84 (1.94–4.17)	<.001	1.23 (0.74–2.06)	0.424
Hypertention				
No	1.00 (Reference)			
Yes	1.79 (0.94–3.40)	0.077		
Diabetes				
No	1.00 (Reference)			
Yes	0.56 (0.30–1.05)	0.072		
Second liver transplant				
No	1.00 (Reference)			
Yes	5.77 (0.60–55.80)	0.130		
Diagnostic				
Malignant liver tumor	1.00 (Reference)		1.00 (Reference)	
Post-hepatitis cirrhosis with decompensation	1.73 (1.22–2.47)	0.002	1.55 (1.04–2.31)	0.031
Metabolic liver disease	1.78 (0.29–10.85)	0.529	0.67 (0.07–6.71)	0.730
Alcoholic liver disease	1.61 (0.68–3.80)	0.280	1.31 (0.51–3.36)	0.577
Others	4.09 (2.19–7.64)	<.001	1.58 (0.70–3.58)	0.269
Surgical procedure				
Living related LT	1.00 (Reference)			
Allogeneic LT	0.72 (0.42–1.23)	0.225		
Homologous split LT	4.32 (0.81–23.17)	0.088		
Meld Score				
≤10	1.00 (Reference)			
11–18	0.83 (0.53–1.28)	0.397		
19–24	1.50 (0.90–2.50)	0.120		
≥25	1.42 (0.92–2.17)	0.111		
Child-pugh Score				
A	1.00 (Reference)			
B	1.02 (0.35–3.02)	0.970		
C	1.30 (0.44–3.83)	0.633		
Biliary complications				
No	1.00 (Reference)			
Yes	0.56 (0.29–1.10)	0.091		
Graft weight				
<1,000 g	1.00 (Reference)			
≥1,000 g	0.79 (0.54–1.15)	0.223		
Cold ischemia time				
<5	1.00 (Reference)			
≥5	0.97 (0.68–1.39)	0.862		
Blood transfusion volume				
0	1.00 (Reference)			
0–1,000	0.78 (0.47–1.29)	0.337		
≥1,000	1.62 (0.96–2.72)	0.069		
Blood loss				
≤1,000 ml	1.00 (Reference)		1.00 (Reference)	
>1,000 ml	1.59 (1.15–2.20)	0.005	1.54 (1.05–2.25)	0.027
Surgery time				
<8 h	1.00 (Reference)			
≥8 h	1.07 (0.77–1.49)	0.675		
Respiratory support				
No	1.00 (Reference)			
Yes	0.88 (0.59–1.30)	0.517		
Unplanned transfer to ICU				
No	1.00 (Reference)			
Yes	1.03 (0.72–1.46)	0.875		
Reoperation				
No	1.00 (Reference)			
Yes	1.36 (0.64–2.90)	0.424		
Early complications				
No	1.00 (Reference)		1.00 (Reference)	
Yes	3.85 (2.68–5.52)	<.001	2.49 (1.66–3.72)	<.001
ASM				
Non-Low ASM	1.00 (Reference)		1.00 (Reference)	
Low-ASM	7.27 (3.60–14.66)	<.001	6.61 (3.08–14.21)	<.001

**Figure 3:**
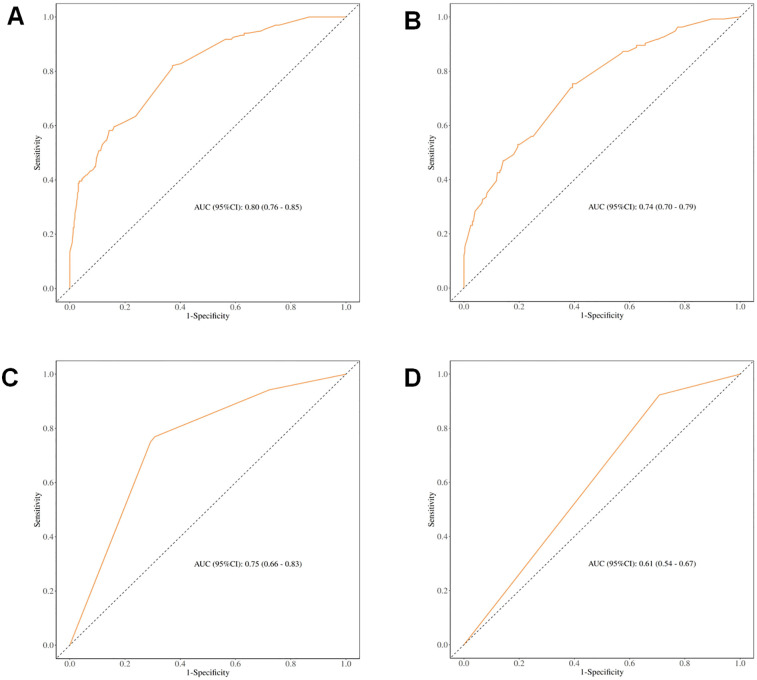
ROC curves for predicting different clinical outcomes. **(A)** ROC curve for early postoperative complications before PSM (including muscle mass); **(B)** ROC curve for early postoperative complications before PSM (excluding muscle mass); **(C)** ROC curve for early postoperative complications after PSM (including muscle mass); **(D)** ROC curve for early postoperative complications after PSM (excluding muscle mass).

Receiver operating characteristic (ROC) curve analysis demonstrated that incorporating ASM into the predictive model significantly improved its performance, with an AUC of 0.80 (95% CI: 0.76–0.85) in the full cohort and 0.75 (95% CI: 0.66–0.83) after PSM. Conversely, the exclusion of the ASM variable diminished the AUC to 0.74 and 0.61, respectively (see Figure 3), underscoring the value of preoperative muscle mass as a predictive marker for postoperative complications.

### 3.4 Correlation between low preoperative ASM and long-term outcomes

**Figure 4:**
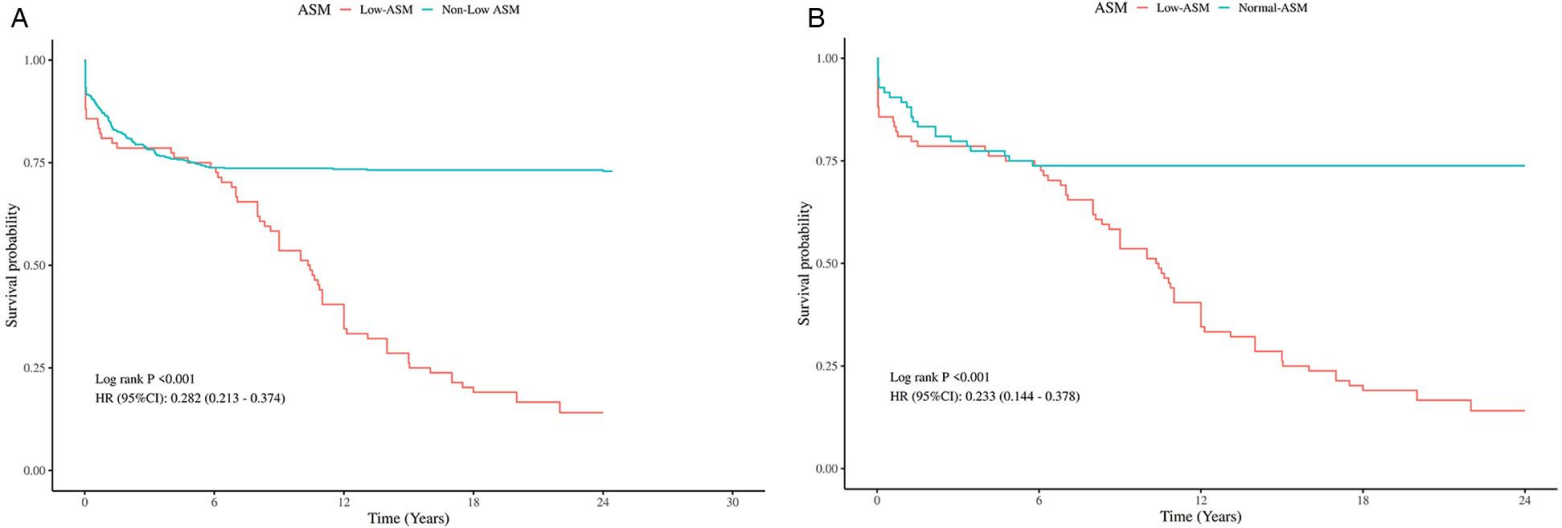
Kaplan–Meier analysis of OS by preoperative ASM Status, Pre- and post-PSM. **(A)** Comparison of OS between patients with and without low preoperative ASM before PSM. **(B)** Comparison of OS between patients with and without low preoperative ASM after PSM.

A total of 631 patients were enrolled in the follow-up cohort, with an average follow-up period of 14.57 ± 9.55 months. Kaplan–Meier survival analysis revealed that, prior to PSM, patients exhibiting low preoperative appendicular skeletal muscle mass had significantly poorer overall survival (OS) than their counterparts without this condition. This difference in survival remained statistically significant even after PSM (Figure 4).

Further analysis using a Cox proportional hazards model demonstrated that a low preoperative ASM was an independent predictor of poor overall survival (HR = 2.25, 95% CI: 1.41–3.57, *P* < 0.001). In addition, intraoperative blood loss greater than 1,000 ml (HR = 1.53, *P* = 0.018) and the presence of early postoperative complications (HR = 2.33, *P* < 0.001) were also significantly associated with worse long-term outcomes (Table 4).

**Table 4:** Univariate and multivariate Cox regression analyses of OS after PSM.

Factors	Univariable analysis		Multivariate analysis	
	HR (95% CI)	*P*-value	HR (95% CI)	*P*-value
Age				
<60	1.00 (Reference)		1.00 (Reference)	
≥60	1.07 (0.70–1.61)	0.002	1.03 (0.66–1.59)	0.903
Sex				
Male	1.00 (Reference)		1.00 (Reference)	
Female	1.70 (1.21–2.39)	0.136	1.02 (0.71–1.47)	0.919
Hypertention				
No	1.00 (Reference)		1.00 (Reference)	
Yes	1.78 (1.04–3.03)	0.034	0.75 (0.39–1.47)	0.409
Diabetes				
No	1.00 (Reference)		1.00 (Reference)	
Yes	0.75 (0.39–1.41)	0.368	0.71 (0.41–1.25)	0.238
Diagnostic				
Malignant liver tumor	1.00 (Reference)		1.00 (Reference)	
Post-hepatitis cirrhosis with decompensation	1.57 (1.12–2.20)	0.010	1.44 (0.99–2.09)	0.055
Metabolic liver disease	1.52 (0.37–6.22)	0.558	0.78 (0.17–3.57)	0.752
Alcoholic liver disease	1.69 (0.77–3.68)	0.188	1.18 (0.51–2.71)	0.697
Others	2.10 (1.26–3.49)	0.004	1.11 (0.61–2.03)	0.738
Surgical procedure				
Living related LT	1.00 (Reference)		1.00 (Reference)	
Allogeneic LT	0.86 (0.51–1.45)	0.576	0.76 (0.38–1.53)	0.442
Homologous split LT	0.89 (0.12–6.69)	0.908	0.36 (0.04–3.03)	0.350
Biliary complications				
No	1.00 (Reference)		1.00 (Reference)	
Yes	0.89 (0.48–1.65)	0.716	0.58 (0.30–1.12)	0.106
Graft weight				
<1,000 g	1.00 (Reference)		1.00 (Reference)	
≥1,000 g	0.93 (0.65–1.35)	0.710	1.15 (0.71–1.86)	0.562
Cooling blood time				
<5	1.00 (Reference)		1.00 (Reference)	
≥5	1.13 (0.79–1.62)	0.501	1.13 (0.76–1.69)	0.536
Blood transfusion volume				
0	1.00 (Reference)		1.00 (Reference)	
0–1,000	0.66 (0.38–1.12)	0.124	0.60 (0.35–1.04)	0.071
≥1,000	1.58 (0.99–2.51)	0.053	1.44 (0.88–2.34)	0.143
Blood loss				
≤1,000 ml	1.00 (Reference)		1.00 (Reference)	
>1,000 ml	1.57 (1.14–2.15)	0.005	1.53 (1.08–2.18)	0.018
Surgery time				
<8 h	1.00 (Reference)		1.00 (Reference)	
≥8 h	1.17 (0.85–1.60)	0.331	1.06 (0.75–1.50)	0.729
Respiratory support				
No	1.00 (Reference)		1.00 (Reference)	
Yes	0.83 (0.57–1.19)	0.304	0.74 (0.49–1.11)	0.144
Unplanned transfer to ICU				
No	1.00 (Reference)		1.00 (Reference)	
Yes	1.17 (0.82–1.65)	0.389	0.84 (0.57–1.25)	0.384
Reoperation				
No	1.00 (Reference)		1.00 (Reference)	
Yes	1.41 (0.77–2.61)	0.267	1.01 (0.51–2.01)	0.968
Early complications				
No	1.00 (Reference)		1.00 (Reference)	
Yes	2.94 (2.16–4.01)	<.001	2.33 (1.58–3.44)	<.001
ASM				
Non-Low ASM	1.00 (Reference)		1.00 (Reference)	
Low-ASM	3.20 (2.28–4.49)	<.001	2.25 (1.41–3.57)	<.001

**Figure 5:**
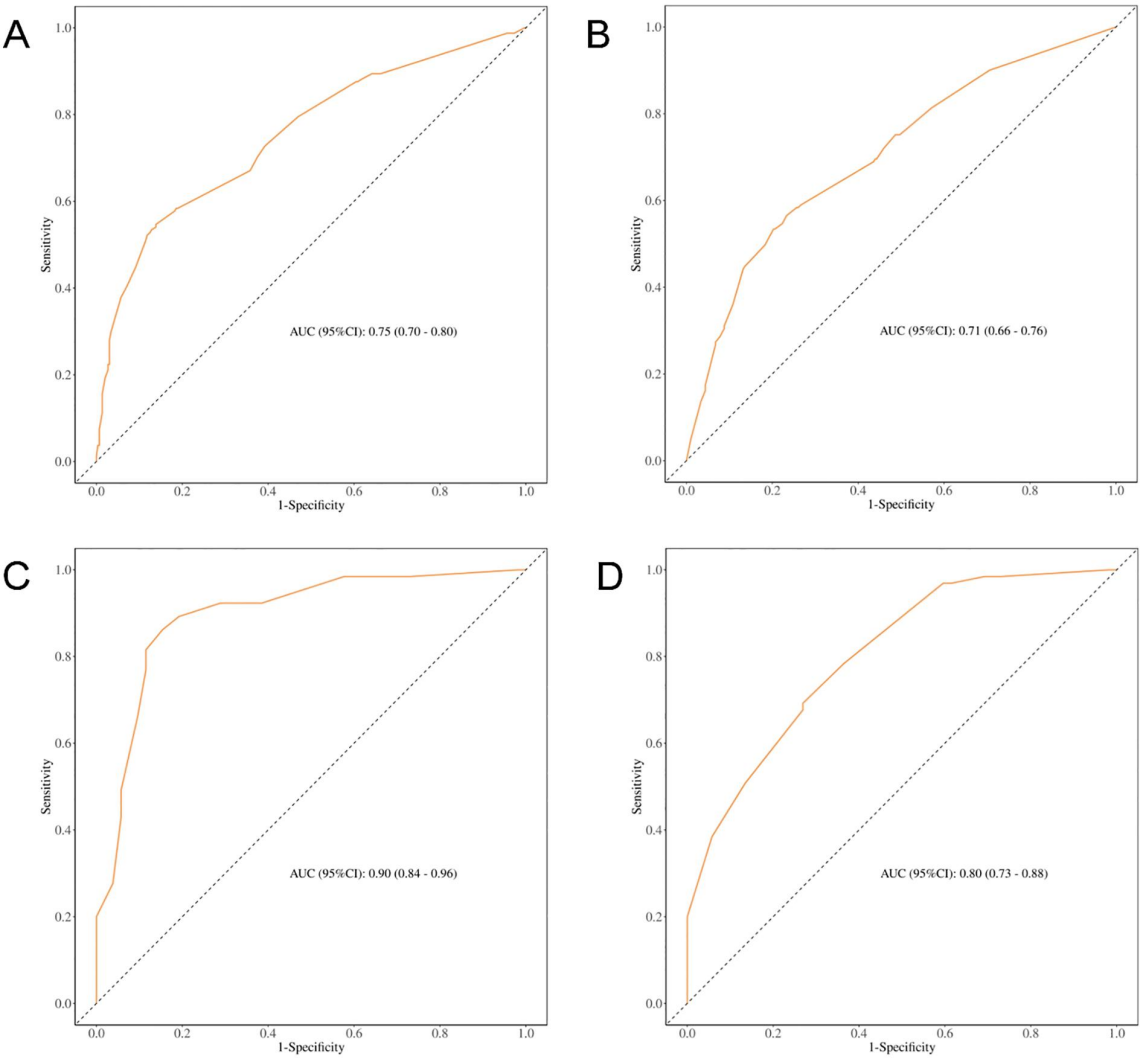
ROC curves for predicting OS based on different clinical models. **(A)** ROC curve for predicting OS before PSM (including ASM); **(B)** ROC curve for predicting OS before PSM (excluding ASM); **(C)** ROC curve for predicting OS after PSM (including ASM); **(D)** ROC curve for predicting OS after PSM (excluding ASM).

ROC curve analysis of various models for OS prediction illustrated an improved predictive capability when ASM was incorporated (see Figure 5), thereby further substantiating the role of skeletal muscle mass as a crucial parameter in preoperative risk assessment.

## 4 Discussion

This study found that among patients who underwent LT between 2015 and 2022, the incidence of a low preoperative ASM was 12.8%. A single-center cohort study from the United States reported a higher incidence of 22%–50% (23), while a Turkish cohort study found a rate of 26% (24), suggesting that the incidence in China is relatively lower but still clinically significant.

Our findings underscore that low preoperative ASM is an independent risk factor contributing to early postoperative complications in liver transplant recipients. After PSM, the incidence of early complications was significantly higher in patients with a low ASM (63.10% vs. 19.05%). These patients are more prone to pulmonary infections, intra-abdominal bleeding, and pleural effusion. Previous studies suggested that postoperative pulmonary infections may be linked to impaired mobility, increased fatigue, and immunosuppression (25–27). The increased incidence of pleural effusion may be secondary to pulmonary infection or associated with hypoalbuminemia caused by malnutrition (28, 29). However, owing to the lack of pleural effusion sample data, it remains unclear whether infection or reduced oncotic pressure is the primary cause, and further investigation is warranted. Postoperative bleeding risk was also notably higher in patients with reduced muscle mass, possibly because of increased portal pressure in patients with sarcopenia (30–32). Overall, these findings suggest that patients with low preoperative ASM are more vulnerable to infections, complications, and delayed recovery following LT. Previous evidence has highlighted the importance of intraoperative blood loss control and nutritional evaluation in improving long-term outcomes (33, 34). Additionally, the study found that patients diagnosed with decompensated cirrhosis before surgery experienced a higher incidence of early complications. Literature has shown that Decompensated cirrhosis is closely associated with muscle wasting, which is driven by impaired nutrient absorption, chronic inflammation, and reduced protein synthesis. In turn, muscle wasting exacerbates liver failure and immunosuppression, creating a vicious cycle (35–37). A meta-analysis by Markakis et al. further confirmed the relationship between preoperative sarcopenia and adverse outcomes (33).

Survival analysis also confirmed that low preoperative ASM was an independent predictor of poor overall ratio [HR] = 2.25, *P* < 0.001). In addition to the increased risk of complications, patients with low ASM have significantly reduced survival benefits. Kalafateli et al. found an association between preoperative sarcopenia and 1-year mortality in 232 liver transplant recipients (38). Esser et al. also showed that patients with low muscle density had higher postoperative mortality (39).

In our cohort of over 600 liver transplant recipients, patients with a low preoperative ASM exhibited a significantly heightened inflammatory response postoperatively. On postoperative days 1 and 3, the neutrophil count, NLR, MLR, and NMR were all significantly higher than those in the non-low ASM group, indicating systemic inflammatory activation. In contrast, lymphocyte and platelet levels were significantly lower, suggesting a weakened immune function. This finding is consistent with the results of previous studies. A retrospective study in Japan reported that preoperative sarcopenia led to an elevated postoperative NLR, both of which are independent predictors of poor prognosis (40). Ding et al. observed increased white blood cells, neutrophils, and SII in patients with low muscle mass (41), whereas Lee et al. showed that combining muscle mass and NLR provided superior prognostic value (42). It is believed that patients with low ASM may exist in a chronic low-grade inflammatory state, with impaired physiological reserves, reduced stress tolerance, and compromised immune function (43, 44). Other studies have proposed that postoperative inflammatory activation may result from aseptic inflammation due to metabolic disturbances or gut microbiota translocation (45, 46). Proinflammatory cytokines such as TNF-u03b1 may also play a role in the pathogenesis of sarcopenia (47). Evidence further indicates that sarcopenia is a potent predictor of sepsis following living donor LT (48). Our findings indicate that both living and deceased donor liver transplant recipients with low ASM exhibit postoperative inflammatory activation, filling a gap in our understanding of systemic inflammation in sarcopenic liver transplant recipients and providing a theoretical basis for postoperative management.

Taken together with the existing literature, ASM appears to be an effective and practical indicator of muscle mass decline. Low preoperative ASM is a strong predictor of postoperative inflammatory activation, increased complications, and reduced survival in liver transplant recipients. Therefore, early identification and management of sarcopenia, including preoperative nutritional interventions, are crucial for improving postoperative recovery.

## 5 Limitations and future directions

This study had several limitations. First, due to its retrospective nature and single-center design, although PSM was used to control for potential confounders, selection bias may still exist. While ASM was shown to have good predictive value for outcomes, sarcopenia is a multifactorial condition also involving physical frailty, fat infiltration, and functional capacity, which were not assessed in this study (8, 49). Furthermore, the presence of ascites and the nutritional condition of patients with liver disease can influence body weight, potentially causing inaccuracies in estimating ASM. Additionally, the unavailability of reliable dry weight measurements or imaging tools like CT or MRI poses a significant limitation to this study. Because of limited data, this study could not thoroughly analyze the link between cold ischemia time and graft function.

The outcome criteria relied on medical record documentation, which might not always align with global standards, indicating that future studies should implement uniform definitions to improve consistency between different centers. We also observed that patients with different liver disease etiologies (e.g., hepatocellular carcinoma, HBV-related cirrhosis, alcoholic liver disease, and autoimmune liver disease) exhibited varying degrees of decompensation and postoperative prognosis. Future studies should investigate whether ASM-based thresholds can be tailored to specific liver disease etiologies.

In addition, future research should consider integrating artificial intelligence (AI) and data lake approaches for liver transplantation research. By leveraging large-scale datasets and advanced AI-driven analytics, it may be possible to generate more comprehensive insights into how different disease conditions and preoperative factors, including ASM, affect post-transplant outcomes. Such approaches could go beyond traditional statistical methods, improving predictive accuracy and facilitating more personalized patient care (50–52).

In essence, ASM serves as a straightforward and effective tool for preoperative risk stratification in LT, potentially aiding intervention strategies. Nevertheless, larger-scale, multicenter prospective studies are indispensable to rigorously validate its predictive accuracy and clinical utility in the liver transplant evaluation process.

## Data Availability

The raw data supporting the conclusions of this article will be made available by the authors, without undue reservation.
